# Understanding the Experiences of COVID-19 Public Health Measures and Well-Being: A Qualitative Study Among Older Adults in Quebec, Canada

**DOI:** 10.1177/10497323241232928

**Published:** 2024-03-05

**Authors:** Julie Karmann, Ingrid Handlovsky, Gregory Moullec, Katherine L. Frohlich, Réjean Hébert, Olivier Ferlatte

**Affiliations:** 1 5622 École de Santé Publique de l’Université de Montréal, Montréal, QC, Canada; 2 Centre de recherche en santé publique, 5622 Université de Montréal et CIUSSS du Centre-Sud-de-l’Île-de-Montréal, Montréal, QC, Canada; 3 School of Nursing, 8205 University of Victoria, Victoria, BC, Canada; 4 Centre de Recherche du CIUSSS du Nord-de-l’Île-de-Montréal (CIUSSS-NIM), Montréal, QC, Canada

**Keywords:** older adults;, COVID-19;, public health measures;, psychological well-being;, ageism;, loneliness;, social cohesion

## Abstract

This interpretative descriptive study explores how public health measures implemented during the first wave of the COVID-19 pandemic in Quebec, Canada, affected the well-being of older adults. Twenty-six participants aged 60–81 took photographs to depict how COVID-19 public health measures affected their well-being and were invited to discuss their photographs in virtual focus groups. Data were analyzed using thematic analysis. The impacts of health measures on the well-being of participants were framed according to three overarching themes. First, participants endured an intensification of ageism, feeling diminished and excluded from their social spheres. Second, they faced a burden of loneliness due to the loss of connections with their communities, particularly for those who were single and without children. Third, participants highlighted navigating a degradation of social cohesion. This manifested through tensions and distrust in both the public and private spheres, as well as acts of resistance in response to rules deemed unjust. While public health measures were essential to prevent onward transmission of COVID-19 and mortality, they negatively impacted older adults’ self-image, loneliness, and trust in society. This study argues for a rethinking of public health norms specific to older adults to address potential sources of inequality. In particular, a greater emphasis is needed on social connectedness and addressing the unique needs of older adults during pandemics.

## Introduction

In response to the rapid surge in COVID-19 cases, the province of Quebec declared a state of emergency on March 13, 2020, and swiftly introduced a series of heath measures, including physical distancing, the closure of non-essential shops and facilities, and a ban on indoor and outdoor gatherings. Recommendations such as wearing masks and confining people over 70 years old were also put into place. Because loneliness was already a major public health issue among older adults prior to the COVID-19 pandemic (Cacioppo & Cacioppo, 2018), significant concerns were raised that confinement and social distancing measures would lead to negative mental health consequences among this population (Armitage & Nellums, 2020; Douglas et al., 2020; Holmes et al., 2020).

As in many other jurisdictions, older adults in Quebec were particularly disadvantaged by the COVID-19 crisis, with age and co-morbidities identified as key COVID-19 mortality risk factors (Santé Québec, 2022). By August 2020, 80% of the mortality related to COVID-19 in Quebec involved people who were living in long-term care facilities dedicated to older adults with impediments or in retirement homes (Public Health Agency of Canada, 2020). Yet, older adults living in long-term care facilities only account for 2.5% of the population over 65 (Commissaire à la santé et au bien-être, 2021), and Quebec’s older adults represent a diverse population, encompassing various profiles in terms of age, culture, job status, family situation, income, place of residence, or health status to name but a few (Fristedt et al., 2021).

Several qualitative studies have investigated the experiences of older adults during the COVID-19 crisis. Findings from these studies have highlighted that the pandemic and its associated public health measures have impacted older adults’ well-being by restricting usual daily activities (Cipolletta & Gris, 2021; Heid et al., 2021; Jimenez-Etxebarria et al., 2021), worsening mental health (aggravated experiences of fear, stress, anxiety, and loneliness) and quality of life (Goncalves et al., 2022; Greenwood-Hickman et al., 2021; Hamm et al., 2020; Xie et al., 2021). Public health measures have also led older adults to negatively change their health behaviours such as becoming more sedentary, eating more often and less healthy, and experiencing disrupted sleep patterns (Bhoyroo et al., 2021; Daly et al., 2021; Greenwood-Hickman et al., 2021; Heid et al., 2021). The public health measures also created important barriers to accessing health-related services and care, further impairing older adults’ well-being (Fiocco et al., 2021). However, the impacts of these measures are dependent on the context and culture in which they are implemented (Flaskerud, 2020). To date, no study has examined older adults’ experiences of public health measures and their consequences on their well-being in Quebec, a culturally unique setting within the Canadian context due to language and cultural differences. Quebec was also subjected to some of the strictest sanitary measures in the country. Gaining insights into the challenges older adults faced during the COVID-19 pandemic can inform public health interventions tailored to meet the unique needs of this group and ultimately enhance well-being.

With the present study, we sought to contribute to and advance understandings of the experiences of COVID-19 of older adults by answering the research question: *How have public health measures affected older adults’ well-being?* We aimed to bring new and nuanced understandings of older adults’ experiences of COVID-19 health measures with regard to their well-being by centring their voices. To do so, we implemented a qualitative study based on photovoice data collection techniques during the first year of the pandemic. In the context where data, and particularly qualitative insights, remain limited, the findings of such work could inform policies and health interventions in future pandemics.

## Methods

### Research Design

Interpretive descriptive methodology (Thorne, 2016) was used to explore older adults’ experiences of the COVID-19 pandemic. Interpretive description is a rigorous qualitative approach with a constructivist orientation that builds upon participants’ experiences and is geared towards generating practice-relevant findings in health disciplines (Thompson Burdine et al., 2021). We combined this approach with data collection techniques adapted from photovoice, a method rooted in social justice and community-based action research where participants take and narrate photographs to illustrate their experiences (Wang & Burris, 1997). We drew on interpretive description and photovoice data collection techniques because we wanted to understand the pandemic from the perspectives of older adults, thereby affirming them as experts on their experiences, and to produce findings that can inform interventions to mitigate the potential harms of public health measures on their well-being. Below, we review the recruitment, sampling, and data collection procedures, which have been documented elsewhere, along with the challenges encountered during the execution of a virtual photovoice as all our research activities were conducted online (Ferlatte et al., 2022). Subsequently, we describe the analytical procedures employed to derive the insights presented in this article. It is noteworthy that a prior analysis of this study delved into the strategies adopted by older adults to enhance their well-being amidst the COVID-19 pandemic (Karmann et al., 2023). In contrast, the current article introduces a fresh analysis of the data, focusing on the adverse consequences of the rapid implementation of public health measures aimed at curbing onward transmission and safeguarding older adults from the COVID-19 virus.

### Recruitment and Sampling

The recruitment strategy was discussed in a committee involving the research team and various community partners from the research team’s network. Following ethics approval from the *Comité* *d’éthique de la recherche en sciences et en santé de l’Université de Montréal*, a flyer describing the study was distributed throughout retirement homes and community groups. These organizations shared the invitation directly with their members in person, in their newsletters and/or on social media. The study inclusion criteria were as follows: (1) being 60 years old or older; (2) speaking and understanding French; and (3) residing in the province of Quebec. As participation in the study was completely online, participants were required to have Internet access and a phone, tablet, or digital camera they could use to take and send photographs. Health status was not considered a recruitment criterion for this study. Potential participants were invited to contact the study team by email or phone. Eligible participants met with a research assistant over Zoom or phone to discuss the project, to receive instructions for taking photographs, and to give oral consent, including consent for the distribution of the images produced. We recruited a total of 26 participants. The study included three subgroups identified in collaboration with our community partners as being particularly at risk of social isolation and other impacts of the COVID-19 pandemic: older adults living alone (*n* = 12), older adults living in retirement homes (*n* = 9), and older adults from the lesbian, gay, bisexual, transgender, and queer (LGBTQ) community (*n* = 5). Most participants were women (*n* = 21) and white (*n* = 25) and age ranged from 60 to 81 years (mean = 71). In total, the 26 participants formed five groups: one group of older adults living in retirement homes, three groups of older adults living alone, and one group of older adults identifying as members of the LGBTQ community.

### Data Collection

Participants were invited to take photographs for a period of 3 weeks to capture their experiences of the COVID-19 pandemic. This included (a) the impacts of the pandemic and the associated health measures on their everyday lives and their well-being and (b) their opinion on the public health measures implemented. Each week, for the entire duration of the data collection (3 weeks), we invited participants to share their photographs with the research team via email or multimedia message service (MMS). During the same week, participants met in an online group to discuss their photographs with other participants. Each discussion groups (*n* = 15) lasted approximately 90 min. The sessions were facilitated by the principal investigator and a doctoral student trained in qualitative research. The photographs were organized into a PowerPoint presentation ahead of the meeting, and each participant was invited to present and describe their photographs. After this, other participants were invited to comment or share whether they had similar or divergent experiences or perspectives. The participants received a $60 honorarium to acknowledge their contribution to the research. The data collection was carried out from May to November 2020.

### Analysis

The focus group discussions were transcribed verbatim and checked for accuracy, and potentially identifying information was removed. Participants were also assigned pseudonyms. Participant photographs were inserted into the transcribed focus group interviews along with the corresponding narratives to be coded and analyzed with the text. NVivo 12 was used to analyze the data, following the steps for thematic analysis described by Braun and Clarke (2006, 2021). First, we read and reread the transcripts to familiarize ourselves with the data, noting our impressions and initial ideas for codes. Second, we performed an initial round of coding to identify and organize the data that were relevant to our research question using process and descriptive coding methods (Saldaña, 2016). Third, after all the transcripts were coded, we adopted a semantic approach where we organized, collated, and summarized the data to identify patterns across the sample related to the impacts of the pandemic. When looking for patterns, we opted for an inductive approach to align with the photovoice aim to understand the viewpoints of community members. Fourth, we reviewed all transcripts to verify the representativeness of the themes. Fifth, we met and exchanged to review and refine the themes, a process that continued throughout the writing of the current article.

## Results

### Enduring an Intensification of Ageism

During the first wave of the pandemic, participants reported feeling stereotyped and discriminated against due to their age, the underpinnings of which stemmed from public health messages that emphasized the vulnerability of older adults to COVID-19 infection. Many participants conceptualized and communicated these experiences as an intensification of ageism, as ageism has been a long-standing experience for many of them.

**Figure 1.**
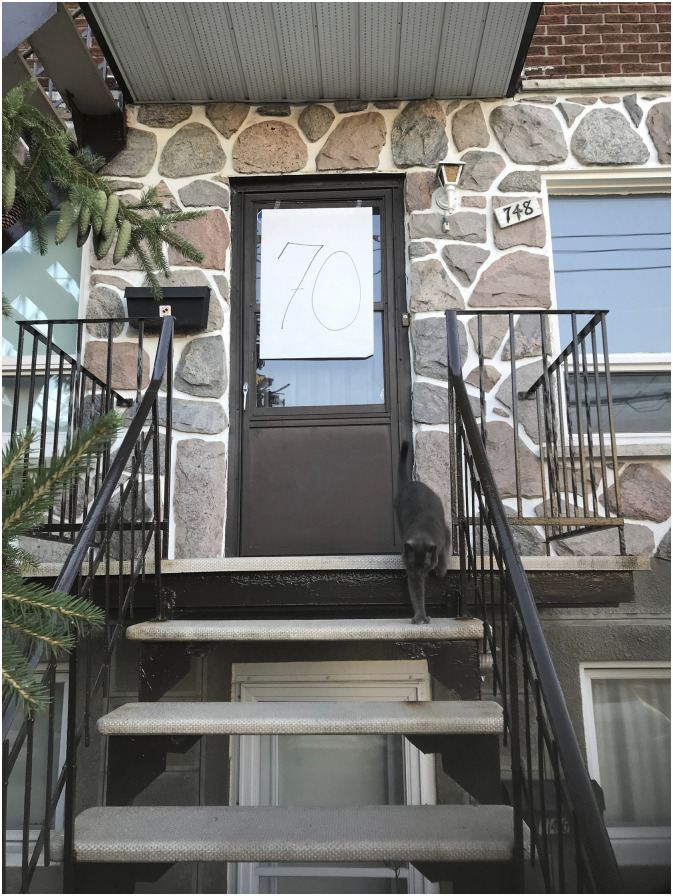
Feeling labelled.

Although participants recognized that the public health measures were intended to protect them, many expressed enduring an intensification of ageism and a resulting diminished sense of individuality – that suddenly they were simply clumped into an age category of ‘older adults’ that diluted any sense of being individuals with unique and diverse attributes. For instance, Marie, who regretted turning 70 years old just before the pandemic, shared a photograph of her front door with the number ‘70’ (Figure 1) which she titled ‘Feeling labelled’. Marie described the photograph as follows: “I thought about putting 70 on the door because I really suddenly thought I was nested, categorized, I could not be an individual person with different aspects to my life, to my personality, to my health.”

Referring to the stay-at-home order for people aged 70 and over, Marie’s words showed how she felt reduced to her age and its associated stereotypes. Similarly, Lise (73 years old) also highlighted that “from 65 years of age to 100 years and over, we are put in a category of elderly … but it’s 35 years of life and there are extraordinary disparities therein!”. Here, Lise emphasized how society was lumping into a single group all older adults, treating them as a monolith and ignoring the huge diversity of experiences, abilities, social conditions, and health statuses among this population. For participants, messages in the media reinforced stereotypes of older adults as frail, vulnerable, lonely, and not contributing to society, which frustrated them as many were involved in their community, working, and volunteering and as such did not identify with the fragile image conveyed in public health messages.

As a consequence, many participants felt a profound sense of social exclusion as they perceived others might have come to associate their age with the COVID-19 virus. This was particularly evident in the narrative of Lise (73 years old) as she described how she felt excluded from her community garden and unwelcomed when grocery shopping due to her age:

… in Montreal what they have done is they have allowed people over 70 years old to have a garden, by putting a red flag in their garden. As if we were lepers … and when people see us coming at the supermarket, at first they looked scared.

This idea of being left out of society and stigmatized was also reflected in Françoise’s (75 years old) perspective as she described how troubled she was by the constant reminders that being an older adult was a risk factor for COVID-19. She argued this led to some negative emotions aimed at older adults:

… the application of the norm that from 70 years and over we were not like the others, we were people who should not be approached and more than that, because it developed a certain hatred towards older people.

Evident in these two testimonies and others were participants’ struggles with consistent messaging about weakness and vulnerability, which they argued was highly stigmatizing and dehumanizing. This resulted in much frustration and an overarching sense of isolation from society as a whole.

Some participants also described how they began to internalize negative messages and stereotypes conveyed about older adults which led to feeling diminished. To illustrate this point, Claire (72 years old) remembered, “…it was the first time I saw myself as old, this was because the government said that I was old and so it changed how I viewed myself a bit.” The feeling of diminishment was all the more present among older adults living in retirement homes where particular health measures were put in place, and these measures were described by many as ‘authoritarian’, pushing the residents into ‘submission’. For example, Robert (75 years old), who was living in a retirement residence with his wife, explained how he experienced the first moment of contact with his family after the beginning of the confinement: “Unfortunately, I had that experience, seeing my grandchildren behind a glass window at the residence. I found it horrible, I found it depressing and humiliating. I felt like a caged animal …” Evident in Robert’s words is the emotional pain he experienced by being deprived of physical contact with his family, which in turn produced the feeling of diminishment.

Overall, despite the well-intended nature of public health measures to protect older adults, they were frequently negatively experienced by participants who described enduring ageism contributing to a feeling of being categorized, socially excluded, and diminished.

### Facing the Burden of Loneliness

While the aim of the COVID-19 public health measures was to prevent onward transmission, the confinement measures led to profound social isolation for older adults. While participants were generally frightened by the prospect of the virus, they were particularly preoccupied by the burden of loneliness that came along with imposed isolation from their communities and families.

**Figure 2.**
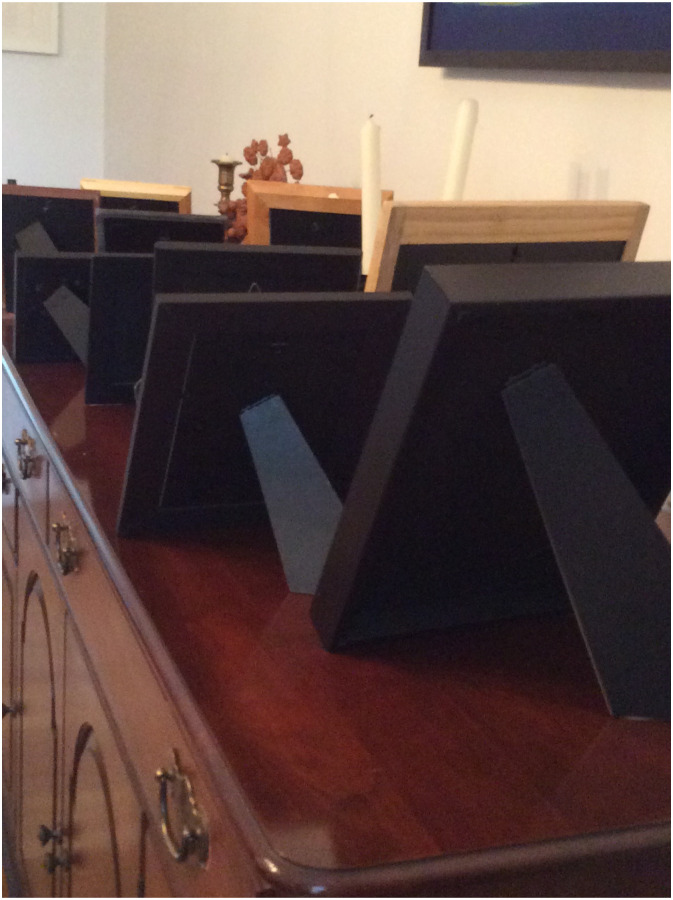
Loss of contact with family.

Participants emphasized the importance of preserving their mental well-being during the pandemic. However, most agreed it was a challenging task given the government-imposed confinement and social distancing measures that led them to be left alone and blocked access to social and support networks, especially their families. Claire (72 years old) who routinely crossed the US–Canada border to visit relatives captured the social distance and her solitude in her photographs showing the back of picture frames of her family members’ photos which she titled ‘loss of contact with family’ (Figure 2). Claire described the photograph as follows:

It’s a part of the structure of my life [traveling back and forth between home and family] and I’ve found it hard not to do that anymore and maybe it’s the hardest part for me. I am completely 100% in agreement that the border is closed, but it’s a little sad … I don’t know.

This testimony acknowledges how, by depriving them of contact with their families, the public health measures disrupted a crucial aspect of enjoyment for older adults.

While many participants expressed loneliness related to weakened family ties, this loneliness could also be the result of a separation from other social circles and individuals the participants interacted with daily. Jacques (65 years old), for example, who lived with his partner, spoke of the void left by the closure of his gym, where he had found a sense of community:

…the gym isn’t just about physical exercise, it’s also about friends, but not close friends. People, men, women with whom we share, we share daily life, we share exercises, we share life. And then from one day to the next you realize that these people, as they are not intimates or friends, you don’t have the contact details of these people, you don’t have this network […] that’s where I felt something was missing.

Evident in Jacques’ testimony was the disruption of the social network related to his sports establishment, depriving him of access to a community where he developed meaningful connections. Like Jacques, many participants reported suffering from the loneliness of being out of touch with communities other than family, such as those related to work, associations in which they were involved, or friends.

Finally, in discussing the mental health impacts of the confinement, some participants revealed that the social isolation imposed by lockdown measures plunged them into a loneliness that dredged up previous memories and experiences such as having been widowed or not having a child. Marie (70 years old), for example, who had been a widow for 17 years, expressed how the pandemic evoked the feeling of nostalgic memories of her life with her husband, which resulted in her feeling extremely isolated: “… I was on my own and I missed having a spouse, I missed my spouse. I had lots of memories of him that unsettled me […]. So, it feels like I’m revisiting my life in a certain way.” Like Marie, other participants found their loneliness amplified because of the weight of the absence of departed loved ones including partners, relatives, friends, or pets. Feelings of loneliness were also amplified, in some cases, among participants who had never had children. Indeed, while the public health measures limited the autonomy of older adults, at the same time public health messages constantly called upon older adults to rely on their families to supply them with basic products – food, medicine, hygiene, clothing, and care items. In these conditions, having no children was sometimes experienced as a feeling of being left all alone in the world, which could lead to panic. Marie (67 years old), who was living in a residence for the elderly and did not have children, remembered the panic she felt when faced with the prospect of not being able to provide for herself while self-isolating for 16 days due to a suspected COVID-19 infection: “But when they asked me that [to remain isolated] and the door closed, I almost panicked at the lack … To say, how would I get supplies?” She further noticed not being the only one in that situation: “But I’ve heard of people here in the corridors who have had outright panic attacks. Because it’s not everyone who has families, children, nephews, who can do their grocery shopping and come and carry them and all that.”

In sum, participants’ narratives illustrate that the public health measures put in place during the first wave of COVID-19 contributed to experiences of loneliness by disrupting and severing much-needed connections with family and community. Furthermore, for those living alone without support, this situation was a major burden, as they felt they could not rely on anyone and were thus reminded of their solitude.

### Navigating a Degradation of Social Cohesion

While the government tried to unite the population around the common enemy represented by the COVID-19 virus, deep ideological divides gradually emerged about the pandemic and the health measures that were swiftly implemented. Participants demonstrated how the ideological divide, through the tensions and mistrust it generated, undermined their sense of social cohesion and how they themselves challenged social cohesion by showing resistance to the adoption of rules they considered unfair.

The ideological cleavage pertaining to the sanitary crisis and the adoption of health measures led to many tensions in the life of participants, affecting their trust in society. Until August 2020, the wearing of masks in indoor spaces was not mandatory in Quebec. As such, challenging circumstances emerged about the dynamics of wearing masks, with some choosing to, while others did not. Participants reported anger against people reluctant to wear masks, which many described led to tense interactions in public places. In particular, the younger generation was often the subject of participants’ grievances, for example, Lise’s (76 years old) statement:

Because I also see that it’s often young people [who don’t respect public health measures]. Because there are some who pass by, they don’t necessarily respect the lines at the pharmacy and all that. […] They don’t respect distance, they don’t think it’s important anyway.

Lise’s testimony describes the stigmatization of younger people, who were mistrusted and perceived as troublemakers and unwilling to do their part to support the safety of others. If the generational divide was obvious in the public sphere, it was also evident within family contexts, igniting conflicts. This was reflected in Marie’s (70 years old) perspective of the new norm of mask wearing as a non-trivial issue linked to strong reactions akin to a political dilemma:

I have children who are completely against the mask. They’re convinced that it’s rubbish, that there’s nothing scientific about it … And I’m at the other end of the spectrum, trying to find the middle ground. I have my own convictions, I wear the mask in public. When we voted yes and no for the PQ [refers here to the referendum for the Quebec’s independency], it was … in the families it was dividing, well, that is the same with the mask.

Comparing the choice to wear a mask to the choice for Quebec independence claimed by the Parti Québécois (known as the PQ) political party in the 1990s, Marie’s testimony emphasized the political dimension invoked by the use of masks as protection in the Quebec context. Marie also revealed how older adults sometimes faced stress navigating the situation within their own family. Here, to keep the peace and preserve cohesion, Marie opted to keep quiet.

Even though, at times, older adults chose to acquiesce for the sake of maintaining peace, some situations were deemed in many ways unfair to them. Thus, they decided to resist what they perceived was a regression of their rights, further contributing to the pressure exerted on social cohesion. Some participants chose to make their voices heard and denounced rules they judged stigmatizing. For example, Lise (76 years old) recounted her battle for keeping her spot in her community garden:

All of a sudden we received this famous sanction, that people aged 70 and over were not entitled to have a community garden. So I told myself: no! And I fought and […] It made me feel good!

**Figure 3.**
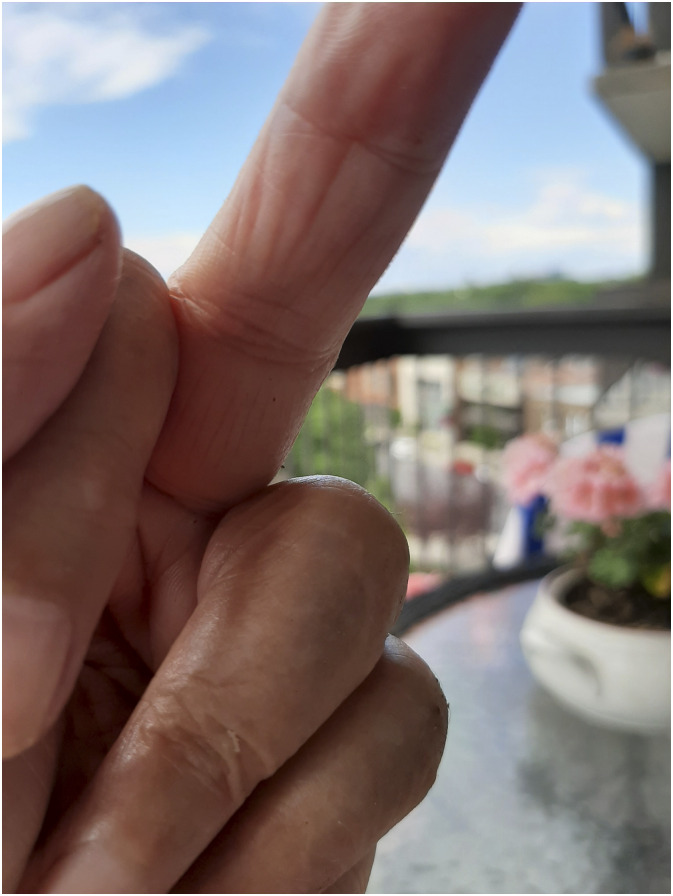
Refusing to be polite.

Instead of adhering to a code of conduct that went against her identity, Lise chose to fight for her well-being and her dignity. She wrote many letters to the authorities to keep the right to enjoy her garden. Faced with the fragility of the older adult care system, as revealed by the high mortality of people living in residential and long-term care centres, many participants advocated for a more decent final stage of their lives. As a result, many were keen to engage in collective struggles so that the well-being of older adults would be advocated for in Quebec. On the other hand, in the face of such a lack of cohesion in society, some participants chose to resist by disinvesting in the collective and turning to more individualistic activities and relying on their own devices. This attitude is anchored in the photo of Lise (76 years old) entitled ‘refusing to be polite’ (Figure 3). Lise, who was living alone in a mixed residence, deviated from the recommendation that people aged 70 and over should not leave their homes. She commented on such a decision: “That was my attitude, that was the attitude of several of my friends who said: fuck! We’re going out anyway!”

This evocative picture, as well as the words of Lise, clearly demonstrates how frustrated participants felt when subjected to rules considered extreme and unjust. This anger and frustration in turn may have led them to be uncooperative, further amplifying the erosion of social cohesion.

In sum, the discretionary nature of some rules eroded the social cohesion of older adults by undermining the well-being and trust of the participants. Public health measures perceived as unfair by older adults drove them to resist, either via collective efforts or through individual acts of civil disobedience, further pressuring social cohesion.

## Discussion

This study sheds light on older adults’ experiences of COVID-19 health measures regarding to their well-being during the first wave of the COVID-19 pandemic in the province of Quebec. The use of photographs combined with discussion groups facilitated a deeper understanding of the intangible impacts of public health measures on older adults’ well-being, namely: (1) enduring an intensification of ageism; (2) facing the burden of loneliness; and (3) navigating a degradation of social cohesion. Understanding these interrelated impacts can guide adjustments to public health measures such that the well-being of older adults is prioritized while minimizing some of the unintended harms associated with the measures.

The first theme, ‘enduring ageism intensification’, echoes recent work (Ayalon, 2020; Derrer-Merk et al., 2022; Falvo et al., 2021; Hafford-Letchfield et al., 2022; Monahan et al., 2020; Nilsson et al., 2021) that has reported an intensification of ageism worldwide as a detrimental side effect of public health measures based on age and not on the health conditions of individuals. Ageism can be defined as “… feeling, belief or behaviour in response to an individual’s or group’s perceived chronological age” (Levy & Banaji, 2002, p. 50). It is an established phenomenon in industrialized societies that has long been socially framed according to people’s age and their corresponding ability to produce capital (Hagestad & Uhlenberg, 2005). Despite being physically protected, participants in this study alluded to other important aspects of public health measures such as their impact on their selfhood. Thus, being diluted in the age-targeted policy and being socially excluded but also internalizing the ageist image led to a feeling of diminution, so to say, feeling ‘othered’ (Cook et al., 2021). Such narratives of negative ageing have health consequences for older adults as they undermine their sense of autonomy and control, create stress (linked to the erosion of self-worth, to the idea of being a burden on society, of not getting adequate care when needed), and contribute to mental health issues (Kessler & Bowen, 2020). Ageism thus represents in itself a costly but overlooked public health problem for a society whose burden is increasing as the population globally ages (Hagestad & Uhlenberg, 2005). As such, ageism requires policies to prevent it, not nurture it.

The second theme, ‘facing the burden of loneliness’, questions the supportive nature of public health measures by prioritizing physical health but severing older adults from social connectedness. Loneliness stemmed from the shattered contacts, not only from family but also with communities (e.g. gym, volunteering, and work) revealing the importance of social connectedness for older adults. Loneliness among older adults as a backlash of public health measures has been widely portrayed in recent publications (Cohn-Schwartz et al., 2022; Falvo et al., 2021; Hayden et al., 2022; Van Tilburg, 2022). Thus, the pandemic exacerbated what had already been recognized as a public health issue prior to the health crisis, in particular among older adults (Holt-Lunstad, 2017). In Quebec, the instruction for those aged 70 and over not to leave their homes has gone hand in hand with the involvement of families in providing for the needs of their elderly. Our study specifically highlighted that in addition to creating a sense of dependency among participants, this measure also led to profound feelings of loneliness among participants who did not meet the social norm of being a parent or having a child to be supported by. The concept of family is a socially constructed phenomenon, and it has experienced changes in the past 50 years. During this period, there has been a noticeable decline in married and partnered individuals, while the number of people living alone has increased (Luhmann et al., 2023; Statistics Canada, 2018). Public health measures and policies should consider these social trends for improved services and interventions.

Lastly, the third theme ‘navigating a degradation of social cohesion’ suggests how sanitary measures created tensions that eroded social cohesion, so to say the norms and attitudes that prevail in a group such as trust and reciprocity (Chan et al., 2006). Ideological cleavages between those who were cautious and those who were not, and particularly old and young, spread from the public sphere into the family unit, leading to stress, struggles, and individual acts of civil disobedience. Our results are in line with those of other work (Brooke & Clark, 2020; Flaskerud, 2020; Greenwood-Hickman et al., 2021; Sipocz et al., 2021). In Quebec, the measures specially targeting older adults, such as the recommendation that people aged 70 and over should remain at home, as well as the non-mandatory mask during the first weeks of the pandemic, and the encouragement of denunciation, have been detrimental to social cohesion, social resilience, and reinforced intergenerational divide (Rocher & White, 2021). Still, in pandemic times, social cohesion is key as it promotes the uptake of public health measures (Borinca et al., 2021) and community resilience (Jewett et al., 2021). Our findings reveal that identity conflicts between generations contaminated the most intimate core for the elderly, namely their families. However, family is the ideal place to recreate intergenerational relationships and help combat ageism (Ayalon et al., 2021). Therefore, it is crucial to rethink the pivotal role that the family should and could play in pandemic times.

From a public health practice perspective, this study advocates for taking into account the diversity and unique needs of older adults in the context of a pandemic. In particular, it emphasizes the need to consider the plurality of realities, capabilities, and needs, not only to combat ageism but also to limit the unintended effects of public health measures, such as loneliness (Ehni & Wahl, 2020). Such a recommendation challenges the current public health culture that too often relies on stigmatizing shortcuts regarding certain populations (e.g. the older adult is vulnerable). Also, given the crucial role that communities – whether related to family, work, neighbourhoods, sports, cultural institutions, or other social circles – play in mitigating loneliness and ageism, it is essential to provide older adults an infrastructure to minimize the consequences of these social disruptions, both in terms of their basic needs and social support. In this sense, digital access for older adults, whether in terms of hardware, literacy, and confidence in one’s ability, appears to be a major public health objective. The implementation of these recommendations could be facilitated by the inclusion of older adults as experts in their communities in the process of co-constructing health measures.

The key strength of this study lies in obtaining first-hand testimonies and harnessing the power of communication technology to give voice to a group of older adults as key informants for improving public health measures. Unfortunately, this study was overly represented by older female adults and older adults younger than 80 years old, suggesting a possible bias. We also acknowledge the selection bias that lies behind such a digitally deployed study. As digital technology turned out to be an essential tool for crisis resilience (Beaunoyer et al., 2020), the impact of the pandemic on older people who did not have access to it undoubtedly would have portrayed a different reality than the one captured by this study. From an equity perspective, further work should be carried out to complete our results with populations of older people not equipped with or able to use digital technology. Additionally, we encountered technical difficulties with the use of Zoom, mainly because our participants were not familiar with this tool. Moreover, we faced some challenges in fostering a positive online group dynamic and building trust. More details about these challenges and how we managed to overcome them are provided elsewhere (Ferlatte et al., 2022). Finally, the study data collection was inspired by the photovoice method (Wang & Burris, 1997) by having participants take and narrate photographs. However, unlike a typical photovoice study (Wang, 1999), participants were not actively involved in the data analysis process. Therefore, despite our intention to empower participants and acknowledge them as experts of their own experiences, the study’s participatory nature is constrained.

## Conclusion

This qualitative study based on data collection techniques adapted from the photovoice method explored older adults’ experiences of COVID-19 health measures with regard to their well-being in Quebec during the first COVID-19 wave. It revealed that public health measures that specifically targeted older adults had a major impact on the perception of ageism. The study results also highlighted that the health measures, through the disruption of relationships they have caused without compensatory measures respecting autonomy and dignity, created an additional health burden for older people in addition to loneliness. Finally, the research showed that discretionary measures, such as the wearing of masks, had a profound impact on social cohesion. The urgency of health situations should not minimize the long-term impacts of the measures put in place, especially when this threatens social cohesion, a factor of resilience in any crisis, which has been unfortunately too often neglected until now. The present work ultimately challenges public health and social norms, sources of inequity, and injustice, particularly concerning older adults.

## Data Availability

The material from which this publication is based as well as the codebook of the analysis can be obtained on request from olivier.ferlatte@umontreal.ca
